# Enhanced antibacterial and anti-biofilm activities of silver nanoparticles against Gram-negative and Gram-positive bacteria

**DOI:** 10.1186/1556-276X-9-373

**Published:** 2014-07-31

**Authors:** Sangiliyandi Gurunathan, Jae Woong Han, Deug-Nam Kwon, Jin-Hoi Kim

**Affiliations:** 1 Department of Animal Biotechnology, Konkuk University, 1 Hwayang-Dong, Gwangin-gu, Seoul 143-701, South Korea; 2 GS Institute of Bio and Nanotechnology, Coimbatore, Tamilnadu, India

**Keywords:** *Allophylus cobbe*, Antibacterial activity, Anti-biofilm activity, Antibiotics, Silver nanoparticles, Sublethal concentrations

## Abstract

Silver nanoparticles (AgNPs) have been used as antibacterial, antifungal, antiviral, anti-inflammtory, and antiangiogenic due to its unique properties such as physical, chemical, and biological properties. The present study was aimed to investigate antibacterial and anti-biofilm activities of silver nanoparticles alone and in combination with conventional antibiotics against various human pathogenic bacteria. Here, we show that a simple, reliable, cost effective and green method for the synthesis of AgNPs by treating silver ions with leaf extract of *Allophylus cobbe.* The *A. cobbe*-mediated synthesis of AgNPs (AgNPs) was characterized by ultraviolet-visible absorption spectroscopy, X-ray diffraction (XRD), Fourier transform infrared spectroscopy (FTIR), X-ray photoelectron spectroscopy (XPS), dynamic light scattering (DLS), and transmission electron microscopy (TEM). Furthermore, the antibacterial and anti-biofilm activity of antibiotics or AgNPs, or combinations of AgNPs with an antibiotic was evaluated using a series of assays: such as *in vitro* killing assay, disc diffusion assay, biofilm inhibition, and reactive oxygen species generation in *Pseudomonas aeruginosa, Shigella flexneri, Staphylococcus aureus*, and *Streptococcus pneumonia.* The results suggest that, in combination with antibiotics, there were significant antimicrobial and anti-biofilm effects at lowest concentration of AgNPs using a novel plant extract of *A. cobbe*, otherwise sublethal concentrations of the antibiotics. The significant enhancing effects were observed for ampicillin and vancomycin against Gram-negative and Gram-positive bacteria, respectively. These data suggest that combining antibiotics and biogenic AgNPs can be used therapeutically for the treatment of infectious diseases caused by bacteria. This study presented evidence of antibacterial and anti-biofilm effects of *A. cobbe*-mediated synthesis of AgNPs and their enhanced capacity against various human pathogenic bacteria. These results suggest that AgNPs could be used as an adjuvant for the treatment of infectious diseases.

## Background

Nanotechnology is a promising field for generating new types of nanomaterials with biomedical applications [1]. Silver nanoparticles (AgNPs) have attracted significant interest among the emerging nanoproducts because of their unique properties and increasing use for various applications in nanomedicine. Silver, in the form of silver nitrate or silver sulfadiazine, has been long used for the treatment of bacterial infections associated with burns and wounds because of its antibacterial properties [2]. Numerous physical, chemical, and biological methods have been developed for the synthesis of AgNPs. However, the synthesis of nanoparticles using conventional physical and chemical methods has a low yield, and it is difficult to prepare AgNPs with a well-defined size [3]. Furthermore, chemical methods make use of toxic-reducing agents, such as citrate, borohydride, or other organic compounds, and can negatively impact the environment. Because the control of particle size and shape is an important factor for various biomedical applications, the use of biological methods to synthesize AgNPs is an environmentally friendly alternative. These methods involve synthesizing AgNPs using bacterial proteins that can exert control over the shape, size, and monodispersity of the nanoparticles by varying parameters such as the type of microorganism, growth stage, growth medium, synthesis conditions, pH, substrate concentrations, temperature, and reaction time [4].

The conventional methods like physical and chemical such as laser ablation, pyrolysis, lithography, chemical vapour deposition, sol-gel techniques, and electro-deposition for synthesis of nanoparticles seem to be very expensive and hazardous. Further, the procedure involves various reactants, in particularly reducing agents (eg., sodium borohydride or potassium bitartrate or methoxypolyethylene glycol or hydrazine) and also it requires a stabilizing agent such as sodium dodecyl benzyl sulfate or polyvinyl pyrrolidone to prevent the agglomeration of metallic nanoparticles. Although many methods are available for the synthesis of nanoparticles, there is an increasing need to develop simple, cost effective, high-yield, and environmentally friendly procedures. Therefore, it is essential to look for alternative green methods for the synthesis of metal nanoparticles [4,5]. In biological methods, a vast array of biological resources easily available in nature including plants and plant products, algae, fungi, yeast, bacteria, and viruses could all be employed for synthesis of nanoparticles, and the time required for complete reduction is lesser. Synthesized AgNPs are readily available in solution with high density and are stable. Among several natural sources, plant and plant products are available easily, and it facilitates synthesis of nanoparticles fairly rapidly. In addition, leaf extracts contain alkaloids, tannin, steroids, phenol, saponins, and flavonoids in aqueous extracts. On the basis of these compounds found in the extracts, we expect that the proteins or polysaccharides or secondary metabolites of leaf extracts can reduce the Ag^+^ ions to Ag^0^ state and form silver nanoparticles. In recent years, various plants have been explored for synthesis of silver and gold nanoparticles. Recently, Singhal et al. [6] synthesized silver nanoparticles using *Ocimum sanctum* leaf extract showed significant antibacterial activity against *E. coli* and *Staphylococcus aureus*. Although several studies have reported the antibacterial activity of silver nanoparticles, the combination of silver nanoparticles and antibiotics studies are warranted.

The increasing prevalence of microbial resistance has made the management of public health an important issue in the modern world. Although several new antibiotics were developed in the last few decades, none have improved activity against multidrug-resistant bacteria [7]. Therefore, it is important to develop alternate and more effective therapeutic strategies to treat Gram-negative and Gram-positive pathogens. Nanoparticles, which have been used successfully for the delivery of therapeutic agents [8], in diagnostics for chronic diseases [9], and treatment of bacterial infections in skin and burn wounds, are one option [10].

AgNPs possess antibacterial [11,12], anti-fungal [13], anti-inflammatory [14], anti-viral [15], anti-angiogenic [16], and anti-cancer activities [17,18]. Developing AgNPs as a new generation of antimicrobial agents may be an attractive and cost-effective means to overcome the drug resistance problem seen with Gram-negative and Gram-positive bacteria. The first aim of the present study was to develop a simple and environmentally friendly approach for the synthesis and characterization of AgNPs using *Allophylus cobbe.* The second aim of this study involved systematically analyzing the antibacterial and anti-biofilm activities of the biologically prepared AgNPs against a panel of human pathogens, including *Pseudomonas aeruginosa*, *Shigella flexneri*, *Staphylococcus aureus*, and *Streptococcus pneumoniae*. The effects of combining antibiotics with AgNPs against Gram-negative and Gram-positive bacteria were also investigated.

## Methods

### Bacterial strains and reagents

Mueller Hinton broth (MHB) or Mueller Hinton agar (MHA), silver nitrate and ampicillin, chloramphenicol, erythromycin, gentamicin, tetracycline, and vancomycin antibiotics were purchased from Sigma-Aldrich (St. Louis, MO, USA). All other chemicals were purchased from Sigma-Aldrich unless otherwise stated. All culture media and chemicals were purchased from Sigma-Aldrich (St. Louis, MO, USA) unless otherwise stated. The strains of *P. aeruginosa, S. flexneri*, *S. aureus*, and *S. pneumoniae* used in the present study were obtained from our culture collection.

### Synthesis and characterization of AgNPs

*Allophylus cobbe* leaves were collected from plants growing in the hills of the Ooty region of India, and stored at 4°C until needed. Twenty grams of *A. cobbe* leaves were washed thoroughly with double-distilled water and then sliced into fine pieces, approximately 1 to 5 cm [2], using a sharp stainless steel knife. The finely cut *A. cobbe* leaves were suspended in 100 ml of sterile distilled water and then boiled for 5 min. The resulting mixture was filtered through Whatman filter paper no. 1. The filtered extract was used for the synthesis of AgNPs by adding 10 to 100 ml of 5 mM AgNO_3_ in an aqueous solution and incubated for 6 h at 60°C at pH 8.0. The bioreduction of the silver ions was monitored spectrophotometrically at 420 nm.

### Characetrization of AgNPs

The synthesized particles were characterized according to methods described previously [4]. The size distribution of the dispersed particles was measured using a Zetasizer Nano ZS90 (Malvern Instruments Limited, Malvern, WR, UK). The synthesized AgNPs were freeze dried, powdered, and used for XRD analysis. The spectra were evaluated using an X-ray diffractometer (PHILIPS X'Pert-MPD diffractometer, Amsterdam, the Netherlands) and Cu-Kα radiation 1.5405 Å over an angular range of 10° to 80°, at a 40 kV voltage and a 30-mA current. The dried powder was diluted with potassium bromide in the ratio of 1:100 and recorded the Fourier transform infrared spectroscopy (FTIR) (PerkinElmer Inc., Waltham, MA, USA) and spectrum GX spectrometry within the range of 500 to 4,000 cm^-1^. The size distribution of the dispersed particles was measured using a Zetasizer Nano ZS90 (Malvern Instruments Limited, UK). Transmission electron microscopy (TEM, JEM-1200EX) was used to determine the size and morphology of AgNPs. AgNPs were prepared by dropping a small amount of aqueous dispersion on copper grids, dried and examined in the transmission electron microscope. XPS measurements were carried out in a PHI 5400 instrument with a 200 W Mg Kα probe beam.

### Determination of minimum inhibitory concentrations of AgNPs and antibiotics

To determine the minimum inhibitory concentrations (MICs) of AgNPs or antibiotics, bacterial strains were cultured in Mueller Hinton Broth (MHB). Cell suspensions were adjusted to obtain standardized populations by measuring the turbidity with a spectrophotometer (DU530; Beckman; Fullerton, CA, USA). Susceptibility tests were performed by twofold microdilution of the antibiotics and AgNPs in standard broth following the Clinical and Laboratory Standards Institute (CLSI) guidelines [19]. The bacterial strains, at mid-log phase (1 × 10^6^/ml), were inoculated into MHB, and 0.1 ml was dispensed per well into a 96-well microtiter plate. *P. aeruginosa*, *S. flexneri*, *S. aureus*, and *S. pneumoniae* were then exposed to different concentrations of AgNPs or antibiotics. Growth was assayed using a microtiter enzyme-linked immunosorbent assay (ELISA) reader (Emax; Molecular Devices; Sunnyvale, CA, USA) by monitoring absorbance at 600 nm. The MICs of AgNPs and antibiotics (Table 1) were determined as the lowest concentrations that inhibited visible growth of the bacteria. Antibiotic or AgNP concentrations that reduced the number of susceptible cells by less than 20% after 24 h of incubation were designated as ‘sub-lethal’ (Table 2). Viability assays were carried out with different concentrations of antibiotics or AgNPs alone, or with combinations of sub-lethal concentrations of antibiotics and AgNPs.

**Table 1:** Determination of MIC value of antibiotics and AgNPs

**Bacterial species**	**Amp**	**Chl**	**Ery**	**Gen**	**Tet**	**Van**	**AgNPs**
*P. aeruginosa*	1.0	2.0	1.0	1.0	1.5	3.0	0.59
*S. flexneri*	1.0	2.0	1.0	1.0	1.5	3.0	0.60
*S. aureus*	2.0	4.0	2.0	2.0	3.0	2.0	0.75
*S. pneumoniae*	2.0	4.0	2.0	2.0	3.0	2.0	0.76

**Table 2:** Determination of sub-lethal value of antibiotics and AgNPs

**Bacterial species**	**Amp**	**Chl**	**Ery**	**Gen**	**Tet**	**Van**	**AgNPs**
*P. aeruginosa*	0.2	0.4	0.2	0.2	0.3	0.6	0.15
*S. flexneri*	0.2	0.4	0.2	0.2	0.3	0.6	0.15
*S. aureus*	0.4	0.8	0.4	0.4	0.6	0.4	2.0
*S. pneumoniae*	0.4	0.8	0.4	0.4	0.6	0.4	2.0

### Disc diffusion assay

The agar diffusion assay was performed as described previously using Mueller Hinton agar [7,12,20]. Conventional and broad spectrum antibiotics were selected to assess the effect of combined treatment with antibiotics and AgNPs. Based on the CLSI standard, the concentrations of antibiotics used were ampicillin (10 μg/ml), chloramphenicol (30 μg/ml), erythromycin (15 μg/ml), gentamicin (10 μg/ml), tetracycline (30 μg/ml), and vancomycin (30 μg/ml). Each standard paper disc was further impregnated with the MIC of AgNPs for each bacterial strain when determining the effects of combination treatments. A single colony of each test strain was grown overnight in MHB on a rotary shaker (200 rpm) at 37°C. The inocula were prepared by diluting the overnight cultures with 0.9% NaCl to a 0.5 McFarland standard. Inocula were applied to the plates along with the control and treated discs containing different antibiotics. Similar experiments were carried out with AgNPs alone. After incubation at 37°C for 24 h, a zone of inhibition (ZOI) was measured by subtracting the disc diameter from the diameter of the total inhibition zone. The assays were performed in triplicate. Antibacterial activity was quantified by the equation (*B* - *A*)/*A* × 100, where *A* and *B* are the ZOIs for antibiotic and antibiotic with AgNPs, respectively [20].

### *In vitro* killing assay

The *in vitro* killing assay was performed as described previously with some modifications [21]. Cells were grown overnight in MHB at 37°C and then regrown in fresh medium for 4 h before being collected by centrifugation and suspended in deionized water. A cell suspension consisting of 10^6^ cells/ml was incubated with various concentrations of antibiotics or AgNPs, or combinations of AgNPs with an antibiotic for 4 h at 37°C. After incubation, bacteria were harvested at the indicated time points and 100-μl aliquots were taken from each sample to determine the number of colony-forming units (CFUs). Experiments were performed with various controls including a positive control (AgNPs and MHB, without inoculum) and a negative control (MHB and inoculum, without AgNPs). All samples were plated in triplicate and values were averaged from three independent experiments. The experiments with sublethal concentrations of antibiotics or AgNPs, or combinations of AgNPs and antibiotics, were performed for 4 h at 37°C.

### Determination of biofilm activity using the tissue culture plate method (TCP)

This assay was performed to determine the ability of AgNPs to inhibit biofilm activity. The assay is based on colorimetric measurements of the crystal violet incorporated by sessile cells [22,23]. Briefly, individual wells of sterile, 96-well flat-bottom polystyrene TCPs were filled with 180 μl of a single bacterial species (1 × 10^6^/ml). After culturing for 24 h, different concentrations of AgNPs were added. The cell culture plates were then incubated for 4 h at 37°C. For combination experiments, bacteria were treated with sublethal concentrations of antibiotics, or individual antibiotics in combination with AgNPs. After incubation, the media were removed and the wells were washed three times with 200 μl sterile distilled water to remove non-adherent bacteria. The wells were air dried for 45 min and 200 μl per well of a 0.1% (*v*/*v*) crystal violet solution in water were added for 45 min. The wells were then washed five times with 300 μl of sterile distilled water to remove excess stain. The dye incorporated by the adherent cells was solubilized with 200 μl of 95% (*v*/*v*) ethanol. The absorbance of each well was measured at 595 nm using a microtiter ELISA reader. The absorbance difference between treated and control wells was considered as an index of bacterial adherence to the surface and thus the activity of biofilms. The percentage inhibition of biofilm activity was calculated using the following equation: [1 - (A_595_ of cells treated with AgNPs/A_595_ of non-treated control cells)] × 100 [24]. Experiments were performed in triplicate. The data are expressed as means ± SD.

### Measurement of reactive oxygen species (ROS) generation

An assay for superoxide anions was carried out according to the manufacturer's instructions (In Vitro Toxicology Assay Kit, (sodium 2,3,-bis(2-methoxy-4-nitro-5-sulfophenyl)-5- [(phenylamino)-carbonyl]-2H-tetrazolium inner salt (XTT) based, catalog number TOX2), was purchased from Sigma-Aldrich, USA*.* All test strains were grown in MHB. Cells were collected, washed with phosphate-buffered saline (PBS), and resuspended in PBS at a concentration of 10^6^ viable cells (determined as CFUs)/ml. XTT was added to the cell suspension at a concentration of 125 μM from a 7.5 mM stock solution in PBS. Cell suspensions were incubated at 37°C on a rotary shaker for 12 h. Aliquots were then removed and spun in a microfuge, and the absorption of the supernatant was measured at 450 nm. The reduction of XTT in the absence of cells was determined as the control and subtracted from the values obtained in the presence of cells.

### Statistical analyses

All assays were carried out in triplicate and the experiments were repeated at least three times. The results are presented as means ± SD. All experimental data were compared using the Student's *t* test. A *p* value less than 0.05 was considered statistically significant.

## Results and discussion

### Synthesis and characterization of AgNPs

Increasing antibiotic resistance is an inevitable consequence of continuous antibiotic usage throughout the world. With the emergence of new virulent pathogens, it is essential to enhance our antibacterial arsenal [21,25]. Recently, there has been significant interest in antibacterial nanoparticles as a means to overcome the problem of drug resistance in various pathogenic microorganisms. Silver ions and salts are known for their potent antimicrobial and anti-biofilm activities. However, although used as a therapeutic agent, silver ions exhibit high toxicity and have relatively low stability because they are easily inactivated by complexation and precipitation with interfering salts [7,23]. To overcome these limitations, we have used an extract of leaf from the *A. cobbe* plant as an environmentally friendly, simple, cost effective, and biocompatible method to synthesize AgNPs.

**Figure 1:**
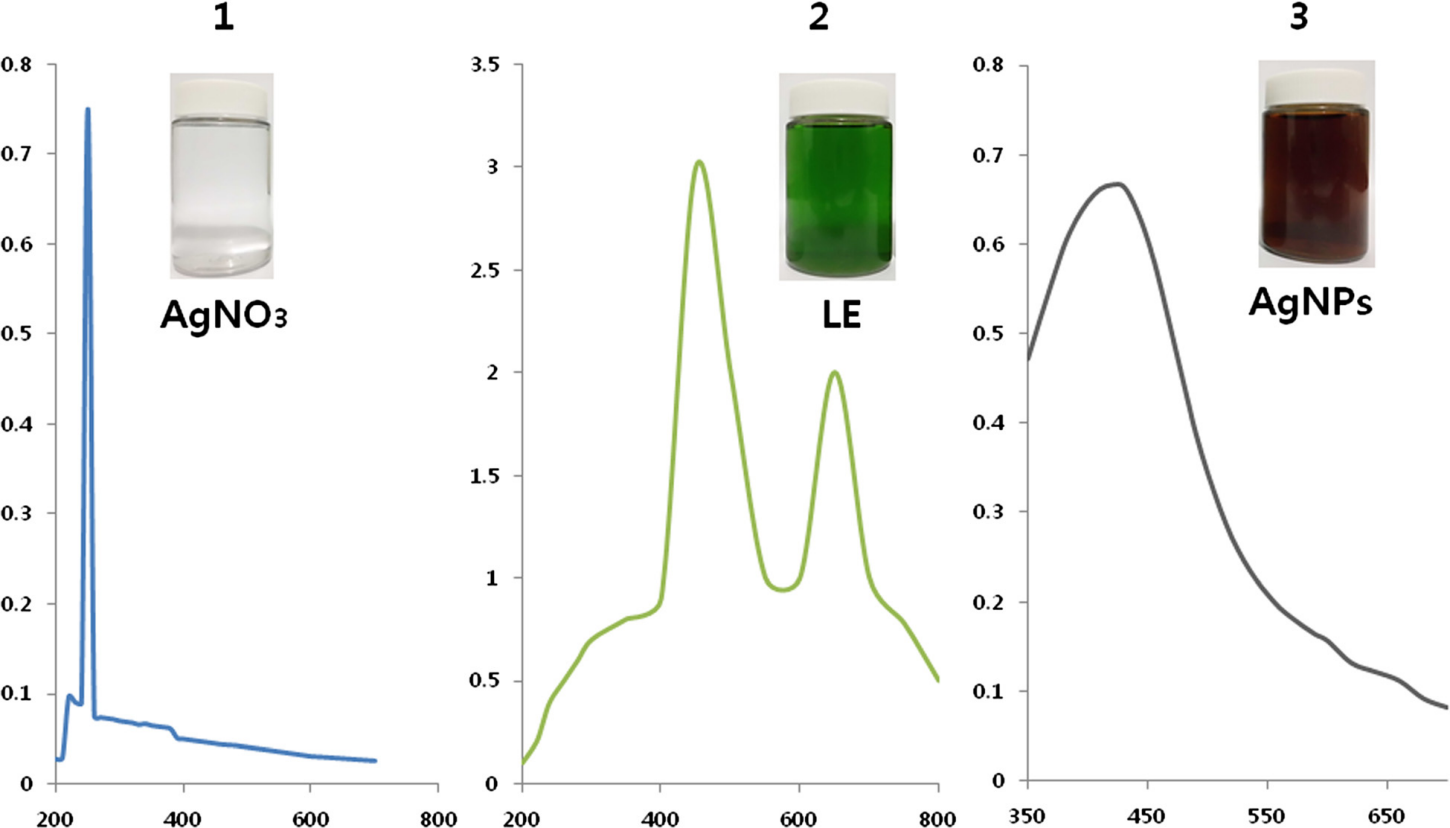
**Characterization of AgNPs synthesized using *A. cobbe *leaf extracts.** The absorption spectra of AgNPs exhibited a strong, broad peak at 420 nm. This band was attributed to the surface plasmon resonance of the AgNPs. The images show the spectrum of AgNO_3_**(1)**, leaf extract **(2)**, and mixture of AgNO_3_ and leaf extract **(3)** at 6 h exposure. After exposure for 6 h, the color of the colloidal solution of AgNPs turned from green to dark brown, indicating the formation of AgNPs.

The aim of this experiment was to produce smaller sizes of AgNPs using *A. cobbe* leaf extract, which acts as a reducing as well as stabilizing/capping agent. In order to control the particle size of AgNPs, 5 mM AgNO_3_ was added to the leaf extract and incubated for 6 h at 60°C at pH 8.0. Synthesis was confirmed by visual observation of the leaf extract and AgNO_3_. The mixture of leaf extract and AgNO_3_ showed a color change from green to brown. No color change was observed during incubation of leaf extract without AgNO_3_ (Figure 1). The appearance of a brown color in AgNO_3_-treated leaf extract suggested the formation of AgNPs (Gurunathan et al. [4,16]; Sathiya and Akilandeswari [26]).

Prior to the study of the cytotoxic effect of AgNPs, characterization of AgNPs was performed according to methods previously described [4]. The synthesized AgNPs were characterized by UV-visible spectroscopy, which has been shown to be a valuable and important tool for the analysis of metal nanoparticles. In the UV-visible spectrum, a strong, broad peak at about 420 nm was observed for AgNPs (Figure 1). The specific and characteristic features of this peak, assigned to a surface plasmon, has been well documented for various metal nanoparticles with sizes ranging from 2 to 100 nm [27,28]. The silver nanoparticles were formed by adding 10 ml leaf extracts with aqueous AgNO_3_. After 6 h, the color of the mixed solutions of leaf extract and AgNO_3_ changed from pale green to deep brown indicating the formation of silver nanoparticles. The change in color of the reaction medium as an effect of presence of reducing potential substances present in the leaf extract. The color of the silver nanoparticles are due to excitation of surface plasmon vibration in silver nanoparticles and this color change is due to redox reaction between the leaf extract and AgNO_3_. AgNPs have free electrons, which give rise to a surface plasmon resonance absorption due to the combined vibration of electrons of the metal nanoparticles in resonance with the light wave. [29] It is observed from Figure 1 that the synthesized AgNPs display a clear and single surface plasmon resonance (SPR) band located at 420 nm which confirms the reduction of silver ion to metallic silver. In contrast, AgNO_3_ shows maximum absorbtion at 220 nm, whereas the leaf extract shows two absorbtion peaks at 450 and 650 nm.The sharp absorption peak of AgNPs indicates that the formation of spherical and homogeneous distribution of silver nanoparticles. The similar observation was reported using leaf extract of *Delonix elata* mediated synthesis of silver nanoparticles [26].

### XRD analysis of AgNPs

**Figure 2:**
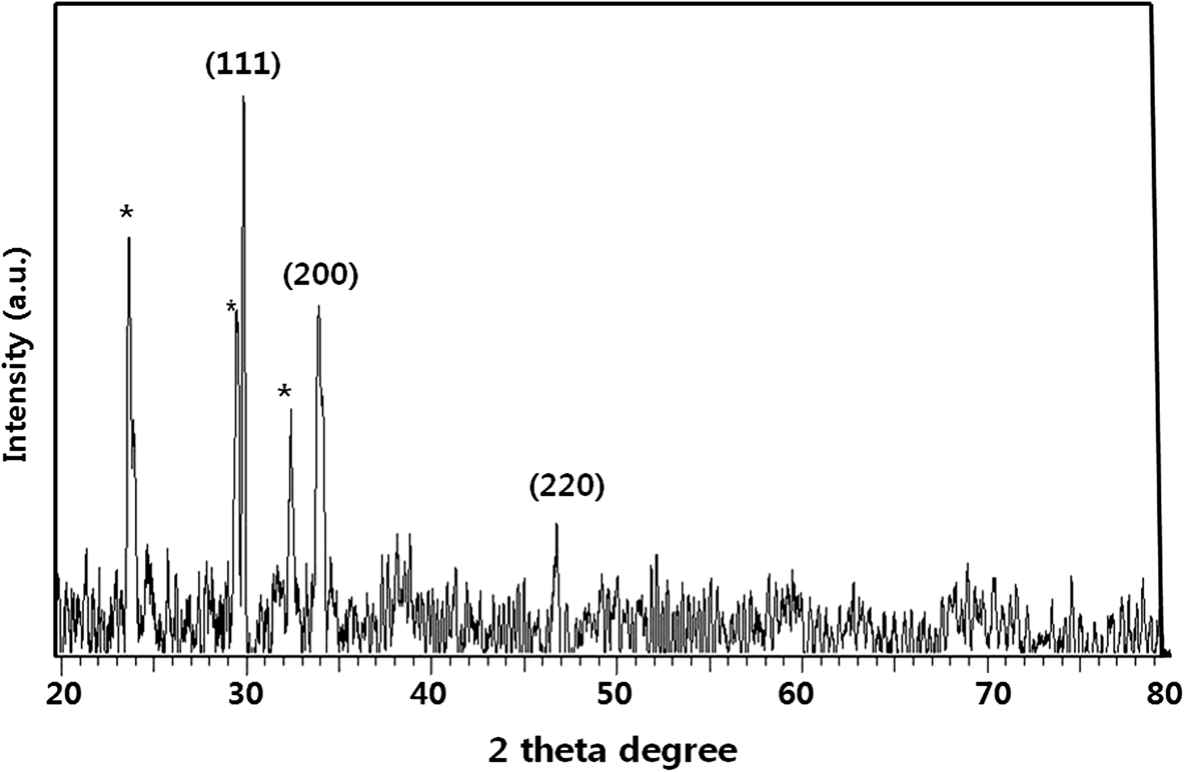
**XRD pattern of silver nanoparticles synthesized using *A. cobbe *** **leaf broth.**

Further, the synthesized silver nanoparticles were confirmed using XRD analysis. Figure 2 shows that the XRD patterns of natural dried silver nanoparticles synthesized using leaf extract. A number of Bragg reflections with 2*θ* values of 24.48°, 30.01°, 33.30°, 34.50°, 46.30° sets of lattice planes are observed which may be indexed to the (111), (200), and (220) faces of silver respectively. The XRD pattern thus clearly illustrates that the silver nanoparticles formed in this present synthesis are crystalline in nature and having face centered cubic (fcc) crystal structure. The XRD pattern confirmed the presence of Ag colloids in the sample. A strong diffraction peak located at 30.01 was ascribed to the (111) facets of Ag. The intensive diffraction peak at a 2*θ* value of 30.01° from the (111) lattice plane of fcc silver unequivocally indicates that the particles are made of pure silver. Two additional broad bands are observed at 34.50°, 46.30° correspond to the (200) and (220) planes of silver respectively (Figure 2). The Braggs reflections were also observed in the XRD pattern at 2*θ* = 24.48° and 32.50°. The assigned peaks at 2*θ* values of 24.48°, 29.0°, and 32.50 (*), may be related to crystalline and amorphous organic phase [26,30-33]. In the obtained spectra, the Bragg peak position and their intensities were compared with the standard JCPDS files. The result shows that the particles have a cubic structure. The size of the silver nanoparticles was found to be 5 nm. The XRD pattern thus clearly indicated that the AgNPs formed in the present synthesis were crystalline in nature. Pasupuleti et al. and other reserachers observed a similar XRD pattern of silver nanoparticles using *Rhinacanthus nasutus* leaf extract. Some unassigned peaks (*) have also been observed suggesting that the crystallization of bio-organic phase [26,30-33].

### FTIR spectra of AgNPs

**Figure 3:**
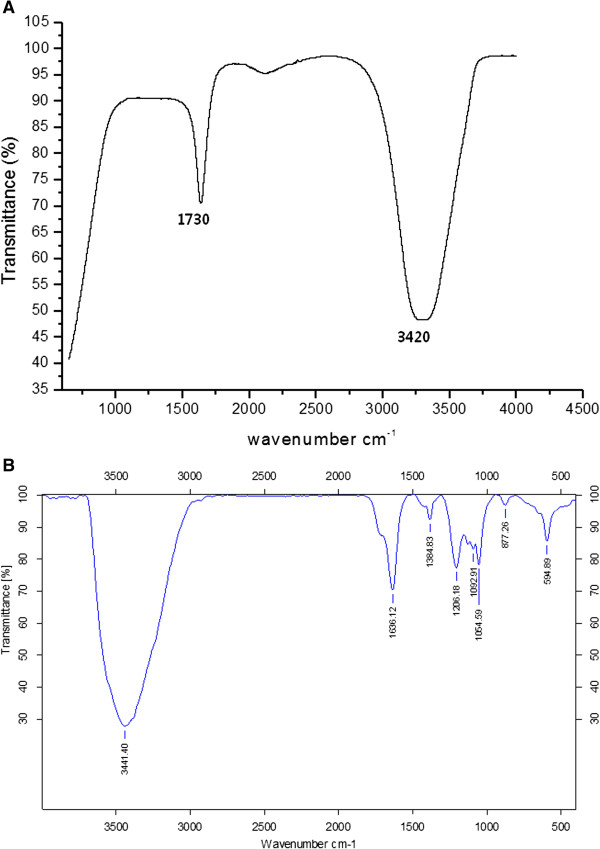
**FTIR spectra of *A. cobbe *** **leaf broth (A), silver nanoparticles synthesized by *A. cobbe *** **leaf broth (B).**

The FTIR spectra were recorded to identify potential biomolecules that contributed to the reduction of the Ag^+^ ions and to the capping of the bioreduced AgNPs [33]. Figure 3A shows FTIR spectra of *A. cobbe* leaf extract observed at 3,420 and 1,730 cm^-1^ are characteristic of the O-H and C = O stretching modes for the OH and C = O groups possibly secondary metabolites of leaf extract. Figure 3B shows the FTIR spectra of purified silver nanoparticles, the presence of bonds due to O-H stretching (around 3,441 cm^-1^), C = O group (around 1,636 cm^-1^), the peak at 1,636 cm^-1^ could be assigned to the vibrations due to amide I band present in the proteins and the peak around 1,384 cm^-1^ assigned to geminal methyl group. The minor band 1,054 cm^-1^ corresponds to C-N stretching alcohols, the band 594, and 887 cm^-1^ regions for C-H out of plane bend, which are characteristics of aromatic phenols [26]. The spectra also illustrate a prominent shift in the wave numbers corresponding to amide I band (1,636 cm^-1^) and amide II band (1520 cm^-1^) linkages, validates that free amino (-NH_2_) or carboxylate (-COO^-^) groups in compounds of the *A. cobbe* leaf extract have interacted with AgNPs surface making AgNPs highly stable. The energy at this vibration is sensitive to the secondary and tertiary structure of the proteins. The band observed at 3,441 cm^-1^ was characteristic of - NH stretching of the amide (II) band. Several bands between 2,000 cm^-1^ to 3,000 cm^-1^ were absent, which could be attributed to protein precipitation occurring during the reduction and stabilization of the AgNPs [33]. We have observed some additional peaks of silver nanoparticles located at around 1,054 cm^-1^ can be assigned as the C-N stretching vibrations of amine. This present result obtained from *A.cobbe* agrees with those reported previously for *Rhinacanthus nasutus*[33], *Thevetia peruviana*[34], latex of *Jatropha curcas*[35]. Our observation lends support to a previous study in which formation of spherical silver nanoparticles was reported by using various plant extracts. Further, the FTIR patterns of *A. cobbe*-mediated AgNPs prove to be powerful evidence in favor of the UV-vis spectra and TEM images for the presence of silver nanoparticles. Several studies have been shown that leaf extracts are responsible for the reduction of silver ions for the synthesis of silver nanoparticles. The absorption peak at 1,636 cm^-1^ is close to that reported for native proteins [36] which suggest that proteins are interacting with biosynthesized nanoparticles. It is well-known that proteins can bind to gold nanoparticles either through free amine groups or cysteine residues in the proteins [37]. A similar mechanism could be possible, the leaf extract from *A. cobbe* cap the silver nanoparticles, thereby stabilizing them. Similar FTIR pattern was also observed for synthesis of silver nanoparticles using Geranium leaf extract [38], *Ocimum sanctum* leaf extract [6,26,39].

### XPS analysis of AgNPs

**Figure 4:**
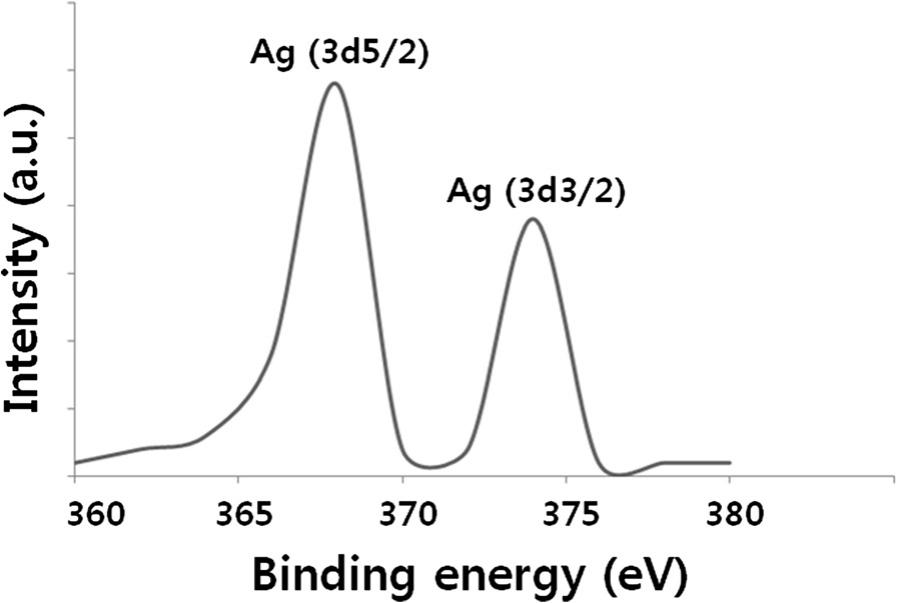
XPS analysis of AgNPs.

X-ray photoelectron spectroscopy (XPS) was utilized to investigate the chemical state of the leaf extract-mediated synthesis of AgNPs. The quantitative Ag/C atomic ratios of the samples were determined using the peak area ratio of the corresponding XPS core levels and the sensitivity factor (SF) of each element in XPS. Figure 4 shows high-resolution XPS spectra of the C(1 s) core level for the AgNPs. The binding energies of Ag(3d5*/*2*)* and Ag(3d3*/*2*)* peaks were found at binding energies of 368.0 and 374.0 eV, respectively. To further understand the chemical state of the AgNPs on the surface, a detailed deconvolution of the Ag(3d) peak was also performed. The binding energy of the Ag(3d5*/*2*)* core level for Ag, Ag_2_O, and AgO is 368.5, 368.3, and 367.7 eV, respectively. Based on the Ag(3d5*/*2*)* peak analysis, we have found that about 93% of the silver atoms on the surface were in the Ag^0^ (metallic) state, while only about 1% and 6% of the silver atoms were in the Ag^+^ and Ag^2+^ chemical states, respectively. These values are in good agreement with published values for AgNPs.

### Particle size distribution analysis of AgNPs

**Figure 5:**
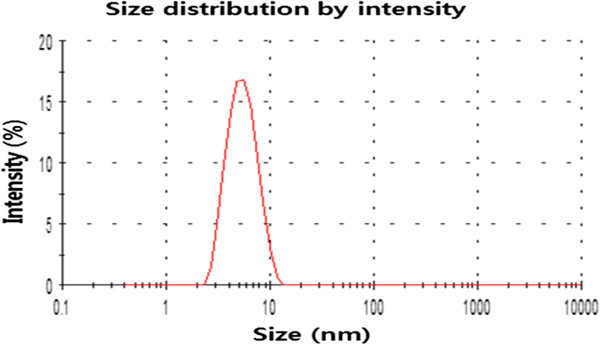
**Size distribution analysis of AgNPs was determined by dynamic light scattering.** The particle size distribution analysis revealed that the average particle size was approximately 5 nm.

TEM images are captured under high vacuum conditions with a dry sample; before analysis of AgNPs using TEM, dynamic light scattering (DLS) was carried out to determine particle size in aqueous solutions using DLS. The characterization of nanoparticles in solution is essential before assessing the in vitro toxicity [40]. Particle size, size distribution, particle morphology, particle composition, surface area, surface chemistry, and particle reactivity in solution are important factors in assessing nanoparticle toxicity [40]. DLS is a valuable technique to evaluate particle size, and size distribution of nanomaterials in solution. In the present study, DLS was used, in conjunction with TEM, to evaluate the size distribution of AgNPs. The AgNPs showed with an average size of 5 nm, which exactly matches with TEM observation (Figure 5). The DLS pattern revealed that leaf extract-mediated synthesized AgNPs showed with an average size of 5 ± 4 nm. Singhal et al. [6] reported that the silver nanoparticles synthesized using *Ocimum sanctum* leaf extract showed an average diameter of 22.38 nm. Recently, Sathiya and Akilandeswari [26] reported that the particle size distribution of silver nanoparticles synthesized by *Delonix elata* leaf broth shows that particles are polydisperse mixture, with average diameter 70.01 nm.

### Size and morphology analysis of AgNPs using TEM

**Figure 6:**
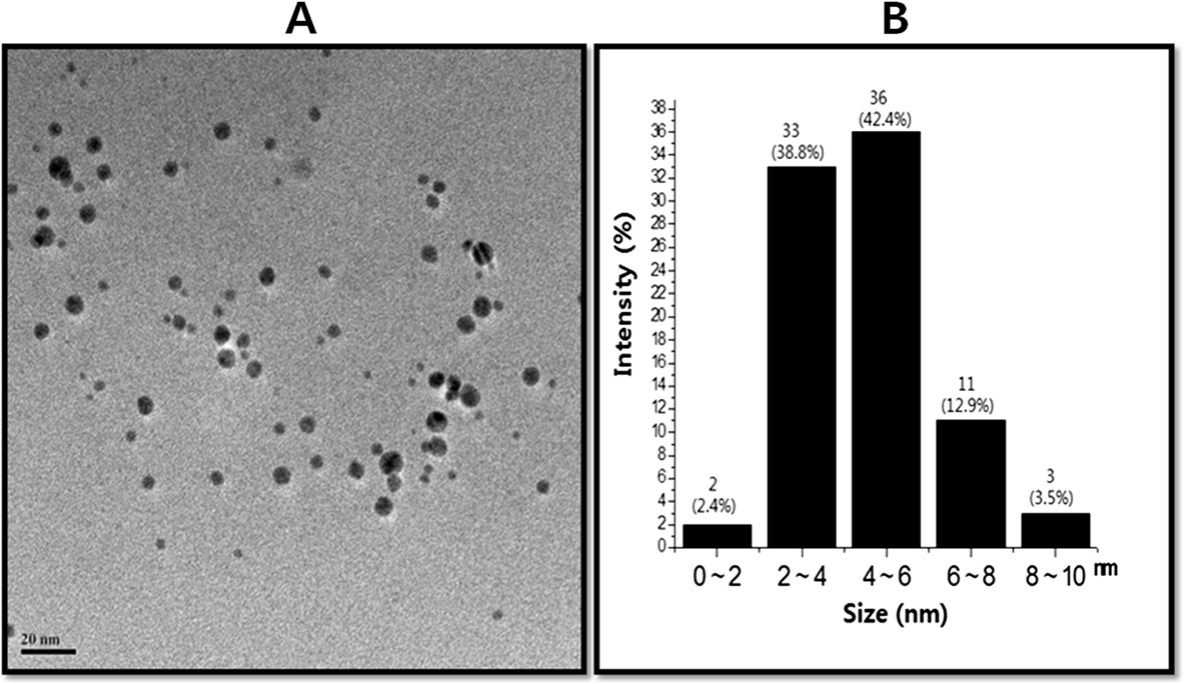
**Determination of size and shape of AgNPs.** The size and morphology of AgNPs were determined using transmission electron microscopy. TEM micrograph of AgNPs prepared from *A. cobbe***(A)**. The average particle size was found to be 5 nm. Particle size distributions from TEM images **(B)**.

TEM is one of the most valuable tools to directly analyze structural information of the nanoparticles. TEM was used to obtain essential information on primary nanoparticle size and morphology [40]. TEM micrographs of the AgNPs revealed distinct, uniformly spherical shapes that were well separated from each other. The average particle size was estimated from measuring more than 200 particles from TEM images, and showed particle sizes between 2 and 10 nm with an average size of 5 nm (Figure 6). Shankar et al. [38] reported that the size of the nanoparticles produced by geranium leaf extract was from 16 to 40 nm. The nanoparticles obtained from leaf extracts of *Catharanthus roseus* showed with an average size of 27 to 30 nm. Rodríguez-León et al. [41] synthesized two different populations of nanoparticles such as small in size with an average diameter around 3 to 5 nm and another one larger in size between 10 to 20 nm using different concentrations of leaf extract and AgNO_3_.

### Determination of MIC and sublethal concentration of AgNPs and antibiotics

The MIC (Table 1) and sublethal concentration (Table 2) of each test strain of bacteria were first determined against antibiotics and AgNPs alone. The results showed that the effective doses were different between Gram-negative and Gram-positive bacteria, with the Gram-negative *P. aeruginosa* and *S. flexneri* found to be more susceptible to AgNPs. In contrast, AgNPs were comparatively less effective against the Gram-positive *S. aureus* and *S. pneumoniae*. This discrepancy could be due to differences in the membrane structure and the composition of the cell wall, thereby affecting access of the AgNPs. The cell walls of both Gram-positive and Gram-negative bacteria have an overall negative charge because of the presence of teichoic acids and lipopolysaccharides, respectively [42]. The potent bactericidal activity of AgNPs against *P. aeruginosa* and *S. flexneri* could be due to strong interactions between cationic plant compounds and the negatively charged cell wall components.

### Dose-dependent antibacterial effects of AgNPs

**Figure 7:**
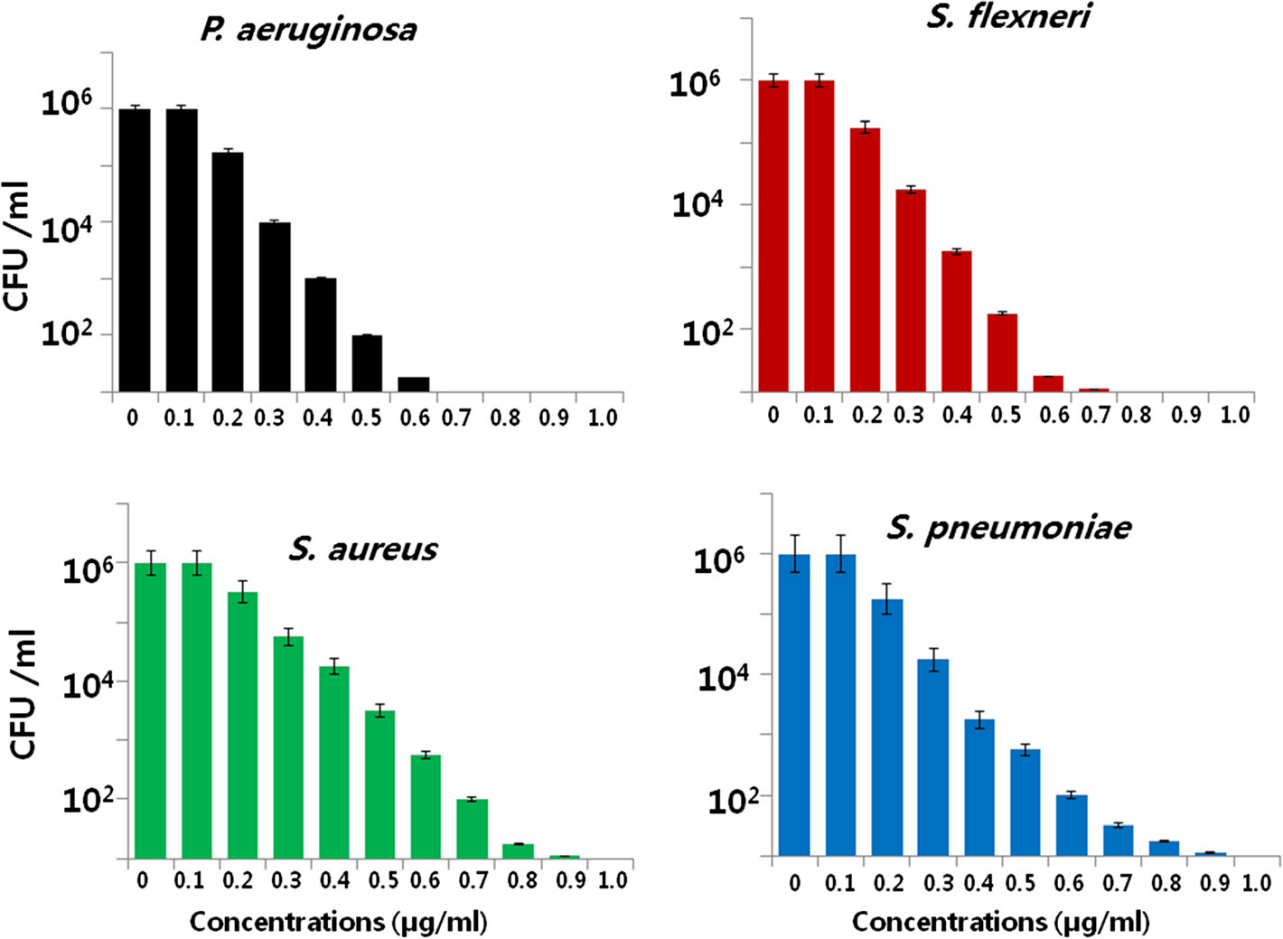
**Effect of AgNPs on cell survival.** Dose-dependent effects of AgNPs on bacterial survival. All test strains were incubated in the presence of different concentrations of AgNPs. Bacterial survival was determined at 4 h by a CFU assay. The results are expressed as the means ± SD of three separate experiments each of which contained three replicates. Treated groups showed statistically significant differences from the control group by the Student's *t* test (*p* < 0.05).

The dose-dependent bactericidal activity of AgNPs was determined using representative Gram-positive and Gram-negative bacterial strains. Figure 7 shows the toxicity of biologically synthesized AgNPs (5.0 nm) at concentrations of 0.1 to 0.6 μg/ml to *P. aeruginosa*, *S. flexneri*, *S. aureus*, and *S. pneumoniae*. The presence of AgNPs affected the cell viability of all bacterial strains as compared to the negative control. Cell viability was reduced as the concentrations of the AgNPs increased. For each bacterial strain, at their respective MIC values, no growth was observed. Thus, these represent bactericidal concentrations for each specific bacterial strain. In the case of *P. aeruginosa*, 0.6 μg/ml AgNPs caused an approximately 95% reduction in bacterial density as compared to the control sample. Increasing the concentration of AgNPs to 0.7 and 1.0 μg/ml caused the complete absence of bacterial growth as these concentrations represent the MIC values. *S. flexneri* showed similar trends with *P. aeruginosa*. Interestingly, for *S. aureus* and *S. pneumoniae,* exposure to a similar concentration of AgNPs (i.e., 0.5 μg/ml) caused a reduction of only about 50% in cell viability as compared to the control sample. However, as the concentration increased to 0.75 μg/ml, there was a much greater inhibition of bacterial growth. The relative order of sensitivity to 5-nm-sized AgNPs was found to be a function of the strain of bacteria.

The plant extract-mediated AgNPs exhibited significant antimicrobial activity than synthesis of AgNPs from other sources such as using bacteria and fungi. For example, Li et al. [43] reported that 10 μg/mL (AgNPs) SNPs could completely inhibit the growth of 10^7^ CFUs/ml of *E. coli* in liquid MHB. Anthony et al. [44] reported that the toxicity AgNPs of size 40 nm was evaluated under non-treated and treated conditions using the cell viability assay; the results showed that 10 μg/ml treatments of AgNPs decreased the cell viability completely. Our studies shows that a promising inhibitory effect of AgNPs against tested strains was observed with lower concentration of 0.6 μg/ml. Hwang et al. [45] reported that chemically derived silver nanoparticles in the size range 10 to 25 nm are effective antimicrobial agents. Earlier studies show that the interaction stage of Ag nanoparticles in *E. coli* and found that at initial stage of the interaction of AgNPs adhere to bacterial cell wall subsequently penetrate the bacteria and kill bacterial cell by destroying cell membrane. AgNPs may pass through the cell wall of bacteria to oxidize the surface proteins on the plasma membrane and consequently disturb cellular homeostasis [46,47]. Several research groups suggested that AgNPs may attach to the surface of the cell membrane and disturb its functions such as permeability and respiration [47,48]. Our results suggest that AgNPs synthesized using plant extract seemed to be smaller in size, which may provide more bactericidal effects than larger particles, as the cellular uptake of smaller nanoparticles is easier than that of larger particles. Altogether, our results suggest that *A. cobbe* leaf extract-mediated synthesis of AgNPs seems to be smaller in size, which is having the larger surface area available for interaction with bacteria and it could provide more bactericidal effect than the larger particles.

### Anti-biofilm activity of AgNPs

**Figure 8:**
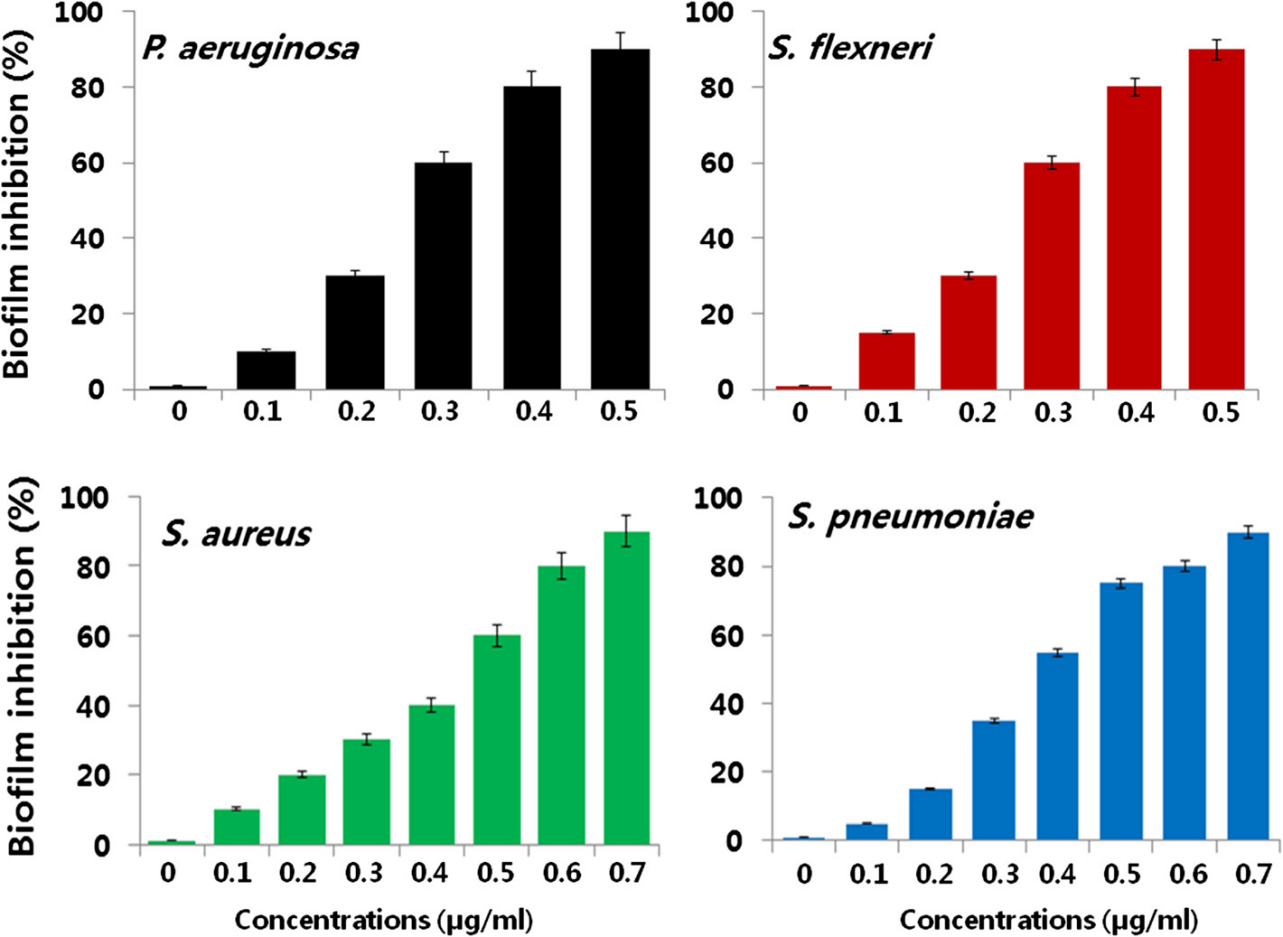
**Effect of AgNPs on biofilm inhibition.** The anti-biofilm activity of AgNPs was assessed by incubating all test strains with different concentrations of AgNPs for 4 h in a 96-well plate. The results are expressed as the means ± SD of three separate experiments each of which contained three replicates. Treated groups showed statistically significant differences from the control group by the Student's *t* test (*p* < 0.05).

AgNPs have been used to inhibit the activity of biofilms. In the current study, the dose-dependent ability of AgNPs to inhibit the activity of biofilms formed by the human pathogens *P. aeruginosa*, *S. flexneri*, *S. aureus*, and *S. pneumoniae* was determined under *in vitro* conditions. All test strains were grown for 24 h in microtiter plate wells and then treated with concentrations of AgNPs of 0.1 to 1.0 μg/ml. These results showed that, for all the tested bacterial strains, the biologically synthesized AgNPs inhibited the activity of biofilms when compared to the negative control (Figure 8). Interestingly, an inhibition of biofilm activity was observed at concentrations of AgNPs slightly lower than those that affected cell viability. Treatment of *P. aeruginosa* and *S. flexneri* for 24 h with 0.5 μg/ml of AgNPs decreased biofilm activity by more than 90%. Although increasing the concentrations of AgNPs did not reveal any significant differences between these two bacteria, treatment of the Gram-positive bacteria *S. aureus* and *S. pneumoniae* with 0.7 μg/ml of AgNPs decreased biofilm activity by approximately 90% (Figure 8). Kalishwaralal et al. [23] reported that anti-biofilm activity of biologically synthesized AgNPs against *P. aeruginosa* and *S. epidermidis* biofilms and found that 100 nM of AgNPs resulted in a 95% to 98% reduction in biofilm formation. Ansari et al. [49] demonstrated that the colonies were grown without AgNPs, the organisms appeared as dry crystalline black colonies, indicating the production of exopolysaccharides, which is the prerequisite for the formation of biofilm, whereas when the organisms were grown with AgNPs, the organisms did not survive. Thus, when the exopolysaccharide synthesis is arrested, the organism cannot form biofilm [49]. Altogether, our data demonstrate that, in these bacteria, the activity of biofilms is more sensitive to AgNPs than is cell death. This suggests that different signaling mechanisms could be involved in cell survival and biofilm formation. Chaudhari et al. [50] reported that AgNPs derived from *B. megaterium* showed enhanced quorum quenching activity against *S. aureus* biofilm and prevention of biofilm formation, and they suggested that AgNPs might be involved in neutralizing these adhesive substances thus preventing biofilm formation.

### Evaluation of enhanced antibacterial effects when combining antibiotics and AgNPs

**Figure 9:**
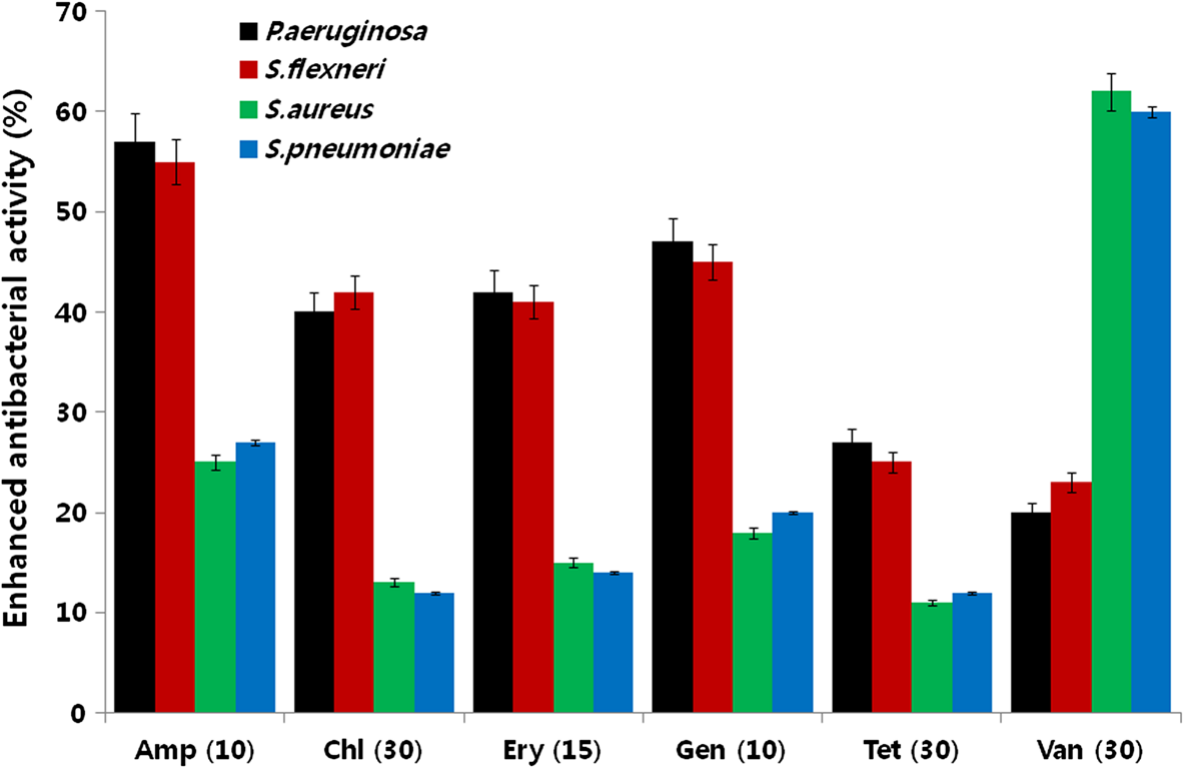
**Enhancement of antibacterial activity of antibiotic in the presence of AgNPs.** Antibacterial activities were determined by the agar diffusion method. The MICs of AgNPs for each test strain were loaded into the wells formed on plates containing a bacterial lawn. Growth inhibition was determined by measuring the zone of inhibition after 24 h. Experiments were performed in triplicate. The percentage of enhanced antibacterial activity was calculated using the formula (*B* - *A*/*A*) × 100. The results are expressed as the means ± SD of three separate experiments. Treated groups showed statistically significant differences from the control group by the Student's *t* test (*p* < 0.05).

The potential additive or synergistic antibacterial effect of combining antibiotics with AgNPs was evaluated using the disc diffusion method. All six antibiotics tested (ampicillin, chloramphenicol, erythromycin, gentamicin, tetracycline, and vancomycin) showed significant (*p* < 0.05) antibacterial effects against both Gram-negative and Gram-positive bacteria (Figure 9). The activities of all the antibiotics were increased in combination with AgNPs in all the test bacterial strains. For the Gram-negative bacteria *P. aeruginosa* and *S. flexneri*, the significant increase in activity in combination with AgNPs was observed for ampicillin (*p* < 0.05). This was followed by gentamicin, chloramphenicol, erythromycin, tetracycline, and vancomycin (*p* < 0.05). In the case of the Gram-positive *S. aureus* and *S. pneumoniae* strains, the order of enhanced sensitivity was vancomycin, ampicillin, chloramphenicol, gentamicin, tetracycline, and erythromycin (all *p* values < 0.05). Ampicillin showed the highest percentage of enhanced activity against both *P. aeruginosa* and *S. flexneri,* and its activity was enhanced by AgNPs. In Gram-positive bacteria, the maximum increase in activity against *S. aureus and S. pneumoniae* was observed with vancomycin. Interestingly, AgNPs increased the susceptibility of all bacterial strains to the antibiotics. These results suggest that there is differential susceptibility between Gram-negative and Gram-positive bacteria to the type of antibacterial agent that is combined with AgNPs. These differences may relate to the cell wall composition of each strain of bacteria.

The toxicity of combinations of antibiotics with AgNPs was assessed by the disc diffusion method. Consistent with earlier reports [12,51], the combined effect of antibiotics with AgNPs was additive. Interestingly, the action of six different antibiotics (ampicillin, chloramphenicol, erythromycin, gentamicin, and tetracycline) showed better enhanced activity against Gram-negative than against Gram-positive bacteria in the presence of AgNPs. There was a significant enhancement seen with ampicillin in *P. aeruginosa* and *S. flexneri* (Figure 9). In contrast, the maximum increase in activity against *S. aureus* and *S. pneumoniae* was observed with vancomycin. These data are consistent with earlier reports [12,51,52]. The differential susceptibility of Gram-negative and Gram-positive bacteria toward antibacterial agents may depend on differences in their cell wall structure [53].

### Enhanced antibacterial effects of antibiotics and AgNPs

**Figure 10:**
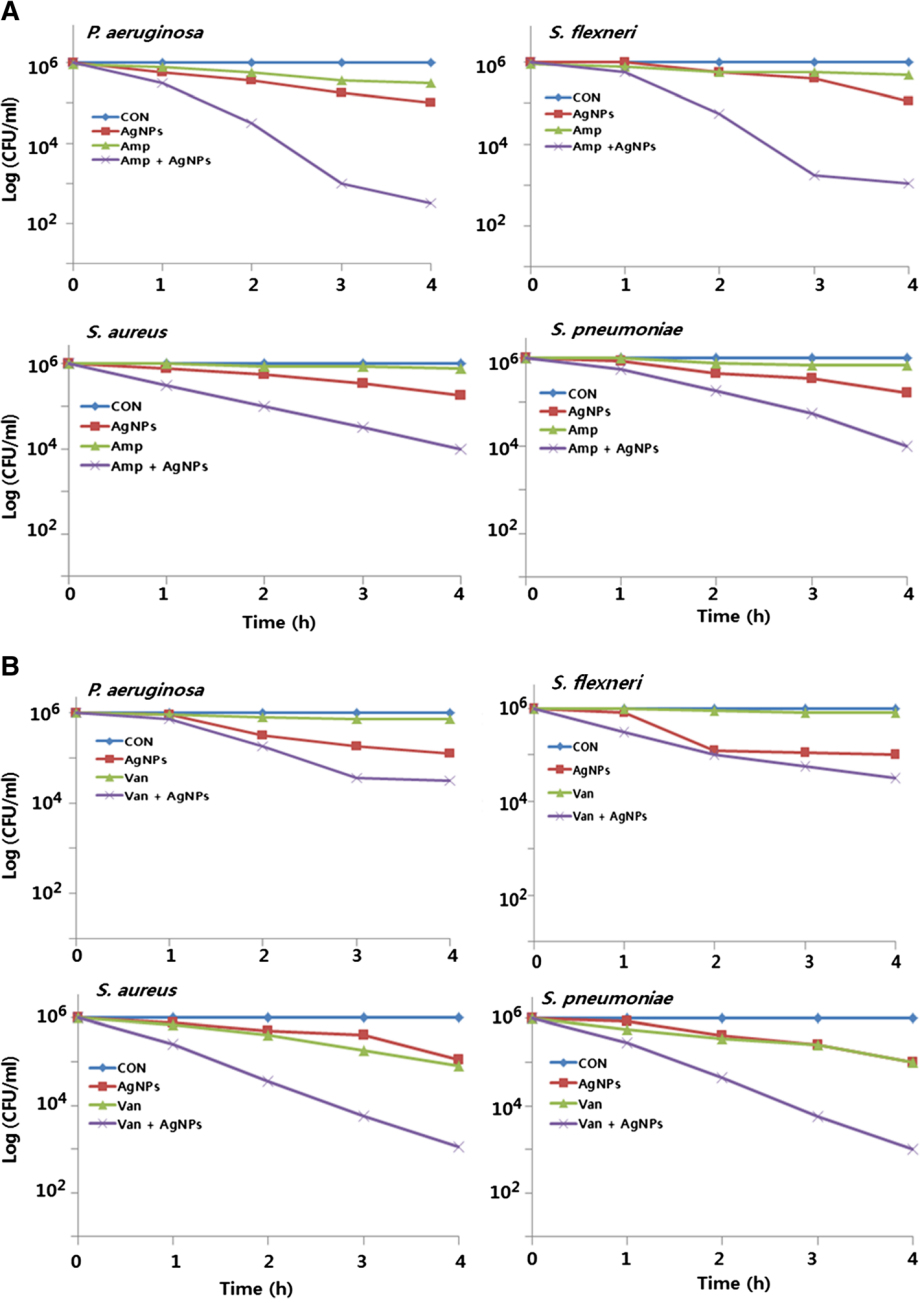
**Enhanced antibacterial effect of antibiotics in the presence of AgNPs.** All test strains were treated for 4 h with sublethal concentrations of ampicillin or AgNPs, or combinations of AgNPs and ampicillin **(A)** or combinations of AgNPs and vancomycin **(B)**. All test strains were treated for 4 h with sublethal concentrations of vancomycin or AgNPs, or combinations of AgNPs and vancomycin. Bacterial survival was determined at 4 h by the CFU assay. The results are expressed as the means ± SD of three separate experiments, each of which contained three replicates. Treated groups showed statistically significant differences from the control group by the Student's *t* test (*p* < 0.05).

*In vitro* killing studies were performed to explore the possibility of using AgNPs as an antibiotic adjuvant, increasing the effect of both AgNPs and antibiotics were analysed using sublethal concentrations. In order to analyze, the bacterial test strains were treated with sublethal concentrations of ampicillin and vancomycin. The addition of sublethal concentrations of AgNPs to these antibiotics treatments resulted in significantly enhanced antimicrobial activity (*p* < 0.05). Interestingly, both of these antibiotics showed an enhanced effect with specific bacteria, compared to control or AgNPs alone. The most significant effects were observed with ampicillin toward Gram-negative bacteria (Figure 10A) and with vancomycin toward Gram-positive bacteria (Figure 10B). Overall, ampicillin displayed significant effects in both Gram-negative and Gram-positive bacteria [18]. A similar inhibitory effect was observed on biofilm activity when these agents were combined. The possibility of using AgNPs as an antibiotic adjuvant [21] was explored by assessing their additive or synergistic effects on bacterial antibiotic susceptibility. The capacity of silver ions to potentiate the bactericidal effect of antibiotics was hypothesized to share a common mechanism of action involving the overproduction of ROS [21,54]. The greatest enhancement by AgNPs was observed with ampicillin against Gram-negative and vancomycin against Gram-positive bacteria. These two antibiotics were, therefore, selected to test the antibacterial and anti-biofilm activity of combined treatments in Gram-negative and Gram-positive bacteria. In this experiment, bacteria were incubated with sublethal concentrations of antibiotics or AgNPs, or combinations of AgNPs and antibiotics, during exponential bacterial growth. CFUs were determined at 24 h after harvesting bacteria at different time points.

The CFU assay showed that sublethal concentrations of antibiotics or AgNPs alone had a killing effect of approximately 10% to 15%. However, combinations of antibiotics with AgNPs resulted in over an 80% decrease in CFUs compared to controls (Figure 10A). Ampicillin exhibited a particularly pronounced antibacterial effect when combined with AgNPs, killing more than 80% of *P. aeruginosa and S. flexneri (p* < 0.05)*.* However, this combination had a much lesser effect on *S. aureus* and *S. pneumoniae.* In response to the combination of AgNPs with vancomycin*,* there was a strong killing effect (*p* < 0.05) on *S. aureus* and *S. pneumoniae* of approximately 78% (Figure 10B). However, this combination showed a much smaller effect on *P. aeruginosa* and *S. flexneri*. These results suggest that, irrespective of the antibiotics, combination treatments resulted in significantly higher toxicity (*p* < 0.05) than in bacterial cells that were treated with AgNPs or antibiotics alone.

### Enhanced anti-biofilm effects of antibiotics and AgNPs

**Figure 11:**
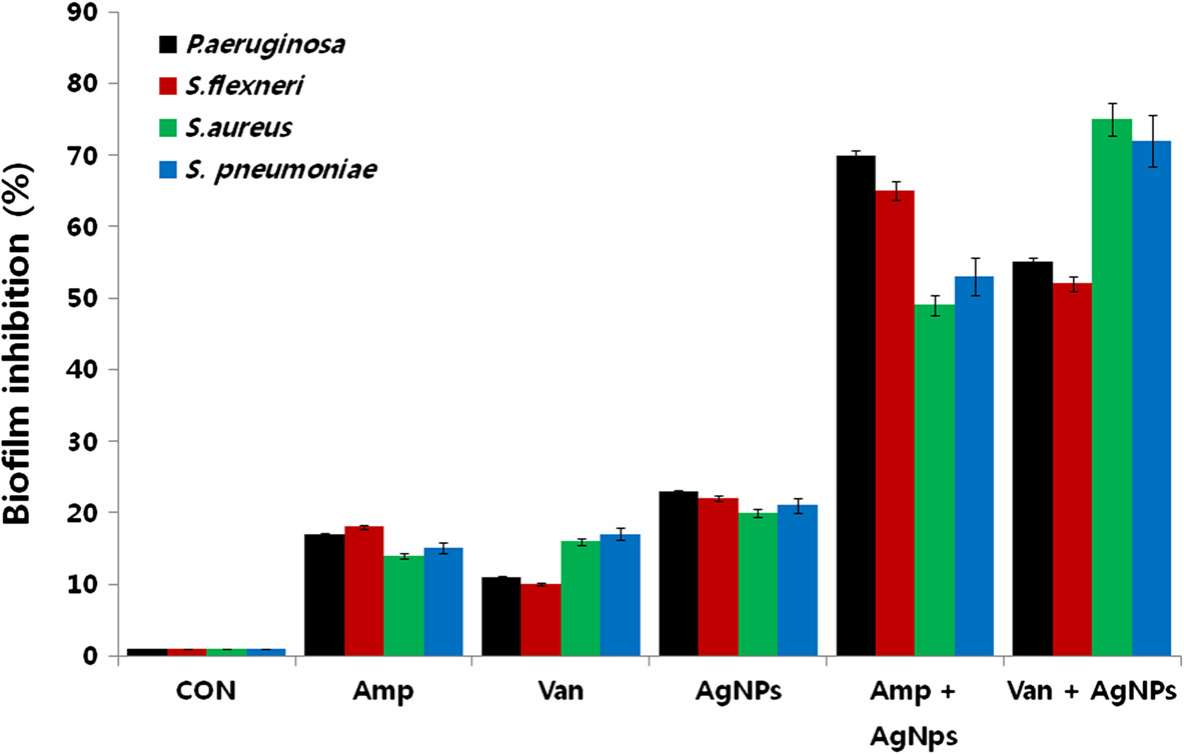
**Enhanced biofilm inhibitory activitity of antibiotics and AgNPs.** The anti-biofilm activity of AgNPs was assessed by incubating all test strains with sublethal concentrations of ampicillin or AgNPs, or combinations of AgNPs with the ampicillin antibiotic for 4 h. The results are expressed as the means ± SD of three separate experiments, each of which contained three replicates. Treated groups showed statistically significant differences from the control group by the Student's *t* test (*p* < 0.05).

Ampicillin has the potential to act at several different stages of biofilm activity with different mechanisms of action [55]. Morones-Ramirez et al. [21] demonstrated, using mouse models, that silver and antibiotic combinations, both *in vitro* and *in vivo,* have enhanced activity against bacteria that produce biofilms. To investigate whether sublethal concentrations of AgNPs in combination with antibiotics have synergistic effects, bacterial cells were grown to form biofilms and then treated with AgNPs alone or in combination with antibiotics. The results indicated that AgNPs alone inhibited biofilm activity by approximately 20%. Combinations of AgNPs and ampicillin inhibited biofilm activity in Gram-negative and Gram-positive bacteria by 70% and 55%, respectively. Combined treatments with AgNPs and vancomycin inhibited biofilm activity in Gram-negative and Gram-positive bacteria by 55% and 75%, respectively (Figure 11). Overall, these data show that combined treatments with AgNPs and antibiotics enhanced both the inhibition of biofilm activity and the levels of cell death. Therefore, combining AgNPs with different antibiotics at lower concentrations has the potential to become an effective anti-biofilm and antibacterial treatment.

The production of biofilms by bacteria can cause resistance to various antibacterial agents. Thus, the inhibition of biofilm activity may be important for the prevention of infections and various other disorders [23]. The ability of AgNPs to inhibit the activity of biofilms was assessed against all of the test strains. There was a concentration-dependent inhibitory effect of AgNPs on biofilm activity (Figure 11). These results showed that treatment with 0.5 μg/ml and 0.7 μg/ml of AgNPs almost completely inhibited the activity of biofilms in Gram-negative and Gram-positive bacteria, respectively. Overall, our results suggest that biologically prepared AgNPs not only exhibit potent bactericidal activity, but also inhibit the activity of biofilms. Our results were consistent with earlier findings suggested that anti-biofilm activity of starch-stabilized nanoparticles in both Gram-positive and Gram-negative bacteria [7].

### AgNPs increases ROS generation in the presence of antibiotics

**Figure 12:**
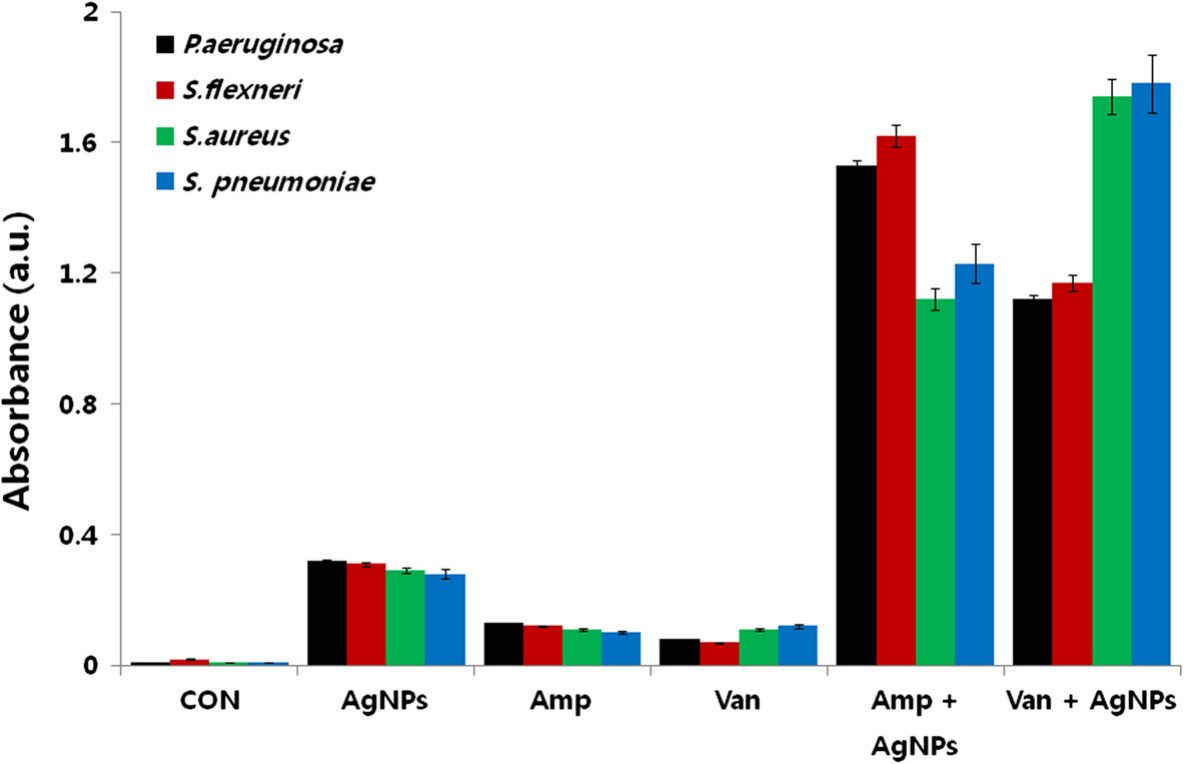
**Enhanced effect of antibiotics and AgNPs on ROS generation.** All test strains were treated with sublethal concentrations of antibiotics or AgNPs, or combinations of AgNPs with antibiotics for 12 h. ROS generation was measured by the XTT assay. The results are expressed as the means ± SD of three separate experiments, each of which contained three replicates. Treated groups showed statistically significant differences from the control group by the Student's *t* test (*p* < 0.05).

The production of ROS, such as hydroxyl radicals, may be a common mechanism of cell death induced by bactericidal antibiotics [21,54,56,57]. AgNPs induce the formation of ROS in several bacterial and mammalian cell types [5]. Several studies have reported that ROS are responsible for inducing genetic variability, promoting or inhibiting cell death, and possibly regulating biofilm development. The current data suggest that sublethal concentrations of antibiotics produce a low level of ROS when compared to AgNPs. The combined treatment of antibiotic and AgNPs showed a significantly higher production of ROS than either agent alone (Figure 12). The moderate level of ROS generated by AgNPs at subinhibitory concentrations could increase membrane permeability and might explain the enhanced activity of ampicillin and vancomycin seen in the presence of AgNPs. As reported previously, increases in ROS production are likely to indirectly affect the interaction of silver with its targets [21].

Cells were treated with sublethal concentrations of antibiotics alone, or in combination with AgNPs. There was a notable increase in the levels of ROS following treatments with AgNPs or antibiotics alone, compared to the control cells. Interestingly, the combined treatment of AgNPs with ampicillin showed a fourfold increase of ROS generation in Gram-negative bacteria (Figure 12). A similar effect was also observed with the combination of AgNPs and vancomycin in Gram-positive bacteria. However, irrespective of the specific antibiotic used, the effect of combined treatments on ROS production was significantly greater than the effect seen with individual agents at subinhibitory concentrations (*p* < 0.05).

Earlier studies demonstrated that improved AgNPs bactericidal activity through silver ion release using nanocomposites [58-67]. It is generally believed that Ag^+^ can bind to bacterial cell wall membrane damage it and so alter its functionality. Ag^+^ can interact with thiol groups in proteins, resulting in inactivation of respiratory enzymes and leading to the production of reactive oxygen species [47,48]. Akhavan [58-60] demonstrated that the main mechanism for silver ion releasing was inter-diffusion of water and silver nanoparticles through pores of the TiO_2_ layer [58]. Akhavan and co-workers demonstrated improved bactericidal activity of the Ni/CNTs and the Ni-removed CNTs by adding silver nanoparticles.

Several studies showed that silver ion release measurements were higher at drying temperature (90°C), which could provide more diffusion of Ag NPs in the porous soft matrix to store a considerable amount of AgNPs in it, resulting in a lasting antibacterial activity [60]. Further, several studies reported that excellent silver ion release in long times through various thin films technologies [60-67].

The mechanism involved in the enhanced antibacterial activity of antibiotics with AgNPs may be attributed to the bonding reaction between nanoparticles and antibiotic molecules. The active functional groups of antibiotics, such as hydroxyl and amino groups, can react with the large surface area of the AgNPs by chelation [51]. Morones-Ramirez et al. proposed a mechanism of silver-induced cell death in which silver may disrupt multiple bacterial cellular processes, including disulfide bond formation, metabolism, and iron homeostasis. These changes may lead to the increased production of ROS and increased membrane permeability that can potentiate the activity of a broad range of antibiotics against Gram-negative bacteria in different metabolic states, as well as to restore antibiotic susceptibility to a resistant bacterial strain. The same mechanism may be at play when using AgNPs as an adjuvant with antibiotics.

## Conclusions

In this work, a systematic methodology was designed to elucidate the enhanced antibacterial and anti-biofilm effects of broad-spectrum antibiotics with AgNPs or without AgNPs. To this end, we synthesized AgNPs using an environmentally friendly approach using supernatant leaf extract of *Allophylus cobbe*. Synthesized AgNPs were then characterized using various analytical techniques. The synthesized AgNPs particles were uniform in size with an average size of 5 nm. Furthermore, the antibacterial activity of the selected antibiotics was increased in the presence of AgNPs against test strains. The increase in activity was more pronounced with ampicillin for Gram-negative bacteria *Pseudomonas aeruginosa* and *Shigella flexneri*; vancomycin for the Gram-positive bacteria *Staphylococcus aureus* and *Streptococcus pneumoniae*. Interestingly, the combination of sublethal concentrations of antibiotics with AgNPs has significantly increased the cell death and increased ROS generation than antibiotics or AgNPs alone. These results could provide a possible mechanism for the synergistic or enhanced effects of antibiotics and AgNPs. These results suggest that AgNPs could be used as an adjuvant for the treatment of various infectious diseases caused by Gram-negative and Gram-positive bacteria. Thus, our findings support the claim that AgNPs have considerable effective antibacterial activity, which can be used to enhance the action of existing antibiotics against Gram-negative and Gram-positive bacteria.

## Competing interests

The authors declare that they have no competing interests.

## Authors’ contributions

SG came up with the idea and participated in the design, preparation of AgNPs, and writing of the manuscript. JWH performed the characterization of nanoparticles. SG, JWH, and DNK participated in culturing, antibacterial activity, anti-biofilm activity, and other biochemical assays. SG and JHK participated in the coordination of this study. All authors read and approved the final manuscript.
